# Publications in Integrative and Complementary Medicine: A Ten-Year Bibliometric Survey in the Field of ICM

**DOI:** 10.1155/2020/4821950

**Published:** 2020-10-06

**Authors:** Zuoqi Ding, Furong Li

**Affiliations:** ^1^School of Science, China Pharmaceutical University, Nanjing, China; ^2^Editorial Department of Chinese Journal of Natural Medicines, China Pharmaceutical University, Nanjing, China

## Abstract

**Background:**

This article aims to analyze the research status of integrative complementary medicine (ICM) and features of highly cited papers in the field to provide reference of the future development of ICM.

**Methods:**

Publications in the field of ICM from 2009 to 2018 were retrieved from the Web of Science Core Collection. The top 20 countries/territories, institutions, journals, keywords of highly cited and noncited papers, and characteristics of essential science indicator (ESI) papers, as well as open access (OA) and non-OA papers, were analyzed.

**Results:**

Mainland China had the largest number of ICM publications. The top 20 journals published a total of 31667 papers in 2009–2018, which represented 92.9% of all publications. Keywords of highly cited and noncited papers point to different research directions. 48 ESI highly cited/hot papers were identified, most of which are related to phytochemistry. Furthermore, the average citation rate (percentage of publications that have been cited one or more times) of OA papers was lower than that of total papers and non-OA papers.

**Conclusions:**

China leads in number of publications; however, publication quality in ICM field requires improvement. A few journals accounted for more than half of number of publications and citations, which are important for the development of ICM. Many of the keywords in ICM noncited publications pointed towards broad meaning that poorly reflect the exact research content. Most highly cited ICM studies focused on the identification and evaluation of plant active components. OA may not be an effective approach to increase paper citations in the field of ICM.

## 1. Introduction

In 2015, the greatest news for scientists in the field of natural medicines, and particularly Chinese scientists, was that Professor Youyou Tu won the 2015 Nobel Prize for Physiology or Medicine, which is the first Nobel Prize in natural sciences awarded to a scientist based on the Chinese mainland. Professor Tu shared this prize (for discovery of avermectins) with Professors William C. Campbell and Satoshi Ōmura, for her unprecedented contribution to medicine that has saved millions of lives worldwide. As declared in her Nobel Lecture on December 7, 2015, at Aula Medica, Karolinska Institutet in Stockholm, the discovery of artemisinin (Qinghaosu in Chinese) is regarded as a gift from traditional Chinese medicine (TCM) to the world. Notably, there is an increasing interest in investigating the biological activities and underlying mechanisms of action of TCM.

The definitions of, and distinctions between, complementary, alternative, and traditional medicine and conventional medicine have been unclear and inconsistent across nations and cultures [1]. Integrative medicine in China always refers to the integration of TCM and Western medicine [2]. TCM is generally considered an important component of integrative and complementary medicine; thus, it is no surprise that scientists from western countries have also contributed considerably to TCM publications [3]. With the development of traditional medicine, combining traditional and modern medicines has been found to show great potential and integrative complementary medicine (ICM) has become an intense focus of new research. ICM has been used for treatment of cardiovascular diseases, cancer, and many other chronic diseases [4–6]. Correspondingly, scientific research output in the ICM field is increasing worldwide.

Moral-Munoz et al. analyzed the research in integrative and complementary oncology from production trends, country collaboration, and leading research topics. The field is led by China, and the primary topics attracting the attention are apoptosis, breast cancer, oxidative stress, chemotherapy, and NF-kappa-B [7]. They also discovered the scientific evolution of cancer-related symptoms in complementary and alternative medicine (CAM) research area and identified the main research thematic by using the coword analysis. The coword analysis identified 12 main thematic areas including anxiety, survivors and palliative care, and meditation [8]. Jose's research studies can reflect the difference between CAM and ICM. Our research will focus on the field of ICM which includes traditional Chinese medicine.

Zhu et al. analyzed the integrated complementary medicine articles based on changes in publication years, geographical distribution, research institutes, and research fields. Their findings show that the development of research and application related to integrated complementary medicine can be divided into three periods. The first period is 2004–2010, which is the period of stable increase. The second one is 2010–2013, which is the period of rapid increase. In the third period, 2014-2015, the number of articles has slightly decreased [9]. We aim to analyze the recent research status of ICM. Considering the delay in the citation of the paper and referring to Zhu's research studies, we set the time as 2009–2018. Zyoud et al. evaluated the performance of research output published in international ICM journals, originating from Arab world. Scientific research output in the ICM field is increasing. Most of publications were driven by societal use of medicinal plants and herbs [6]. We will pay attention to the theme of the highly cited papers in China.

Fu et al. analyzed the quantity and citation impact of scientific papers in the field of CAM. This study suggested that the major type of document is original article. The CAM papers are mostly published by North America, East Asia, and European countries. Major contributors of CAM papers are from USA, China. International coauthorship increased rapidly [10]. Moral-Muñoz et al. presented a keyword analysis of 18,536 articles published in 21 journals in the WoS subject category Integrative and Complementary Medicine from 1974 to 2011 and demonstrated that the study of medicinal plants is a growing area of research, with a good rate of publication and citation of published documents [11]. We will focus on the topics of highly cited papers in the field of ICM and analyze the phytochemistry-related publications in it.

Danell analyzed the reception of ICM research outside the ICM context such as in general or specialized medicine journals. A majority of the cited documents were acknowledged in journals and documents that could be related to research areas outside the ICM context, such as pharmacology and pharmacy and plant science. The related ICM research was related to basic preclinical research, in fields such as cell biology, plant pharmacology, and animal experiments [12]. We will pay attention to the journals of highly cited papers in the ICM field.

Bibliometrics, first introduced by Pritchard, is an effective method that uses quantitative analysis and statistics to describe research trends in a specific field. Indeed, bibliometric approaches have been widely applied to analyze the development of various scientific fields [13–15]. In the present study, we conducted a bibliometric analysis of ICM research from 2009 to 2018. Our results provide a basis for better understanding of global ICM research development.

## 2. Methods

### 2.1. Search Strategy

Using the search strategy: Web of Science (WoS) category, Integrative and Complementary Medicine, publication year 2009–2018, and publication type “article” and “review,” publications in the field of ICM were retrieved from the WoS Core Collection; data were collected in November 2019. Then, the data set is imported into the InCites database for analysis. According to the WoS classification criteria, journals classified in the ICM category were within the scope of the search. The WOS subject classification is many-to-many. A journal can belong to multiple WOS subject classifications at the same time.

A total of 34103 publications were identified. Publications in our research only referred to articles and reviews; other types of publications were not included in our research.

### 2.2. Statistical Analysis

Statistical Product and Service Solutions (SPSS) software was used to analyze correlations among indicators. When analyzed the research topics of the top 500 highly cited and noncited papers (randomly selected by year), we extracted the author keyword and ID keyword plus standard in WoS for comparative analysis. Different words with identical meanings were grouped and considered as a single keyword.

**Figure 1:**
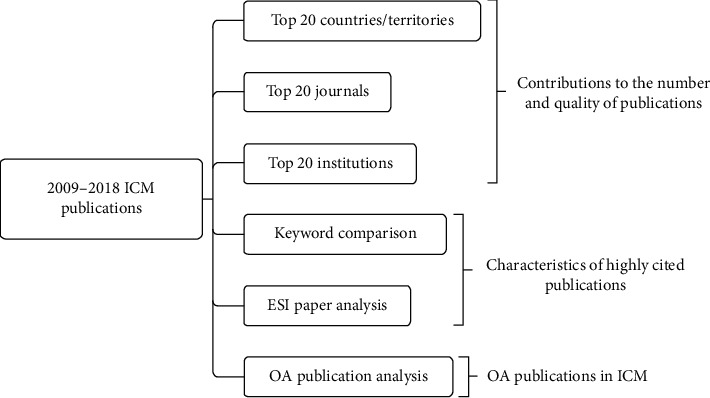
Flowchart of analysis conducted in this study.

An analysis flowchart is presented in Figure 1. We selected the top 20 countries/territories, institutions, and journals for detailed comparisons using multiple bibliometric indices.

An analysis flowchart is presented in Figure 1. We analyzed the top 20 countries/regions, institutions, and journals that contributed most to the quantity and quality of publications in the ICM field. Multiple bibliometric indices were used for detailed comparisons through keyword comparison and Essential Science Indicator (ESI) paper analysis to determine the characteristic of highly cited papers in ICM field. Finally, the impact of open access on the citation of papers in the ICM field was discussed.

## 3. Results

**Figure 2:**
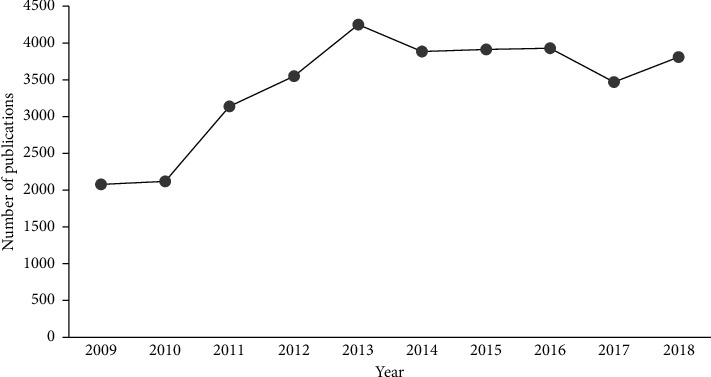
Trend in the number of publications in ICM.

Number of publications is the most unambiguous indicator of scientific output. From 2009 to 2018, 34103 publications in the field of ICM were indexed in the WoS Core Collection database. In 2009, 2076 publications were published, while in 2018, the number of publications had increased to 3797, indicating a rapid increase in number of publications in the field of ICM. There was a clear change in trajectory in 2013, whereas number of ICM publications grew slowly before that point and then gradually reached a steady state in the years following 2013 (Figure 2).

### 3.1. Publication Distribution in the Top 20 Countries/Territories during 2009–2018

**Figure 3:**
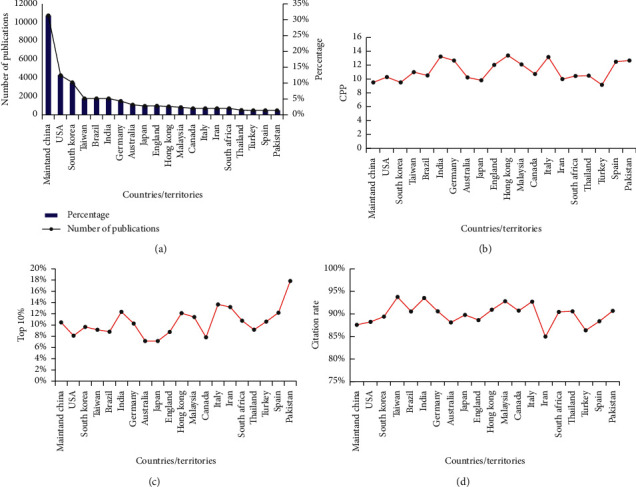
Top 20 most active countries/territories according to number/percentage of publications (a), citations per paper (CPP) (b), top 10% (c), and citation rate (d) during 2009–2018.

According to the author information from the 34103 ICM publications published between 2009 and 2018, a total of 164 countries/territories were involved. The top 20 countries/territories for publication output are shown in Figure 3(a). China ranks first in the number of publications (10672, 31.55%), followed by the United States (4270, 12.62%), South Korea (3445, 10.18%), Taiwan (1733, 5.12%), Brazil (1730, 5.11%), and India (1698, 5.02%). In view of its long history of clinical use of ICM, it is not surprising that China is most active, as evidenced by the number of publications. Notably, ICM research in several other countries/territories also appears to be flourishing, with the numbers of publications increasing during 2009–2018.

The mean number of citations per paper (CPP) is an indicator that is used to compare the scientific impact of publications among countries, institutions, and journals [16, 17]. Although the number of publications from mainland China was highest (rank 1), the CPP (9.51) was somewhat lower than the global mean level (10.22). Among the top 20 countries/territories, 14 countries had higher CPP than the global mean, including the USA, Taiwan, Brazil, India, Germany, England, Hong Kong, Malaysia, Canada, Italy, South Africa, Thailand, Spain, and Pakistan (Figure 3(b)).

Waltman and Schreiber introduced an approach to calculate percentile-based indicators. Their research shows the nice property of percentile-based indicators: a (small) change in the number of citations of one or more publications that are exactly at the top 10% threshold affects only research groups that have publications at the threshold. Research groups without publications at the threshold are not affected [18].

Pakistan's top 10% indicator ranks first, accounting for 17.87%, far higher than Italy, which ranks second, accounting for 13.66%, and other countries account for about 10%. At the same time, considering the number of Pakistani publications is 431, ranking 20th, it is not difficult to conclude that most of the ICM publications in Pakistan have received more citations. We further analyzed the research area of the publications in Pakistan, we found that, in addition to being classified into the field of ICM, 162 publications were classified into Pharmacology & Pharmacy category. Chemistry, Medicinal, and Plant Sciences categories have 148 publications, respectively. At the same time, most of the top 50 highly cited publications in Pakistan are related to the pharmacological effects of plant extracts. The total number of publications in China is far ahead, but the top 10% indicator is not prominent, indicating that many publications published by China in ICM have not received enough citations (Figure 3(c)).

The citation rates of the top 20 countries/territories were in the range of 85%–94%. The correlation between citation rate and CPP (*r* = 0.575) indicates that cited papers may greatly facilitate an increase in CPP (Figure 3(d)).

### 3.2. Publication Distribution of the Top 20 Institutions in 2009–2018

**Figure 4:**
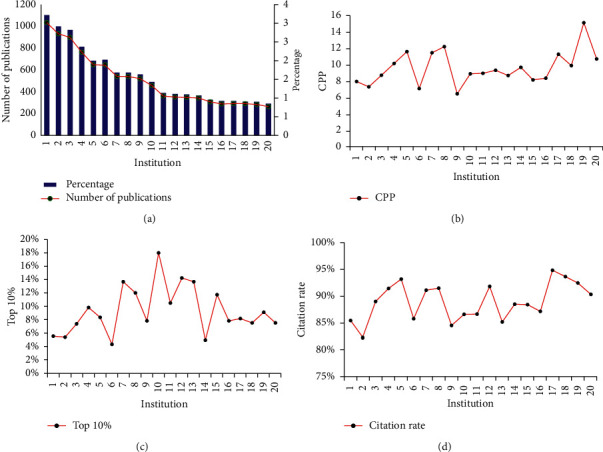
Top 20 institutions according to numbers/percentages of publications (a), citations per paper (CPP) (b), top 10% (c), and citation rate (d) during 2009–2018. (1) China Academy of Chinese Medical Sciences. (2) Beijing University of Chinese Medicine. (3) Kyung Hee University. (4) China Medical University, Taiwan. (5) Shanghai University of Traditional Chinese Medicine. (6) Korea Institute of Oriental Medicine (KIOM). (7) Nanjing University of Chinese Medicine. (8) Chinese Academy of Sciences. (9) Guangzhou University of Chinese Medicine. (10) China Pharmaceutical University. (11) China Medical University Hospital, Taiwan. (12) Chinese Academy of Medical Sciences, Peking Union Medical College. (13) Chinese University of Hong Kong. (14) Capital Medical University. (15) Tianjin University of Traditional Chinese Medicine. (16) Fudan University. (17) Peking University. (18) University of California System. (19) Zhejiang Chinese Medical University. (20) Pusan National University. Mainland China: 1, 2, 5, 7, 8, 9, 10, 12, 14, 15, 16, 17, and 19. South Korea: 3, 6, and 20. Taiwan: 4 and 11. Hong Kong: 13. USA: 18.

Among the top 20 institutions for number of publications, 13 were in mainland China, one was in Hong Kong, three were in South Korea, two were in Taiwan, and one is in the USA. China Academy of Chinese Medical Sciences, Beijing University of Chinese Medicine, and Shanghai University of Traditional Chinese Medicine ranked top 3 for number of publications (Figure 4(a)). The global baseline CPP was 10.76, and the CPP of five institutions from mainland China, Hong Kong, and Taiwan was above the global baseline (Figure 4(b)). The top 10% indicator of China Pharmaceutical University is outstanding, indicating that the papers published by China Pharmaceutical University in the field of ICM have received more citations (Figure 4(c)).

The Chinese University of Hong Kong had the highest CPP. Citation rates of the top 20 institutions ranged from 82.3%–94.9%. Consistent with these findings, we observed a good correlation between citation rate and CPP (*r* = 0.772) (Figure 4(d)).

### 3.3. Publication Distribution of the Top 20 Journals in 2009–2018

The number of journals publishing ICM papers was relatively stable across the period analyzed. Thirty-seven journals published ICM articles and reviews in 2009–2018. Since 2009, the numbers of journals were 27, 25, 27, 28, 28, 28, 27, 28, 28, and 26 per year.

ICM publications are published in relatively concentrated journals. We analyzed the top 20 journals by number of publications. In 2009–2018, the top five journals published more than half of total publications, with the top ten journals accounting for more than 70%, and the top 20 journals accounting for more than 90%. Hence, a few journals hosted more than half of publications and citations in the field, which is important for the development of ICM.

**Figure 5:**
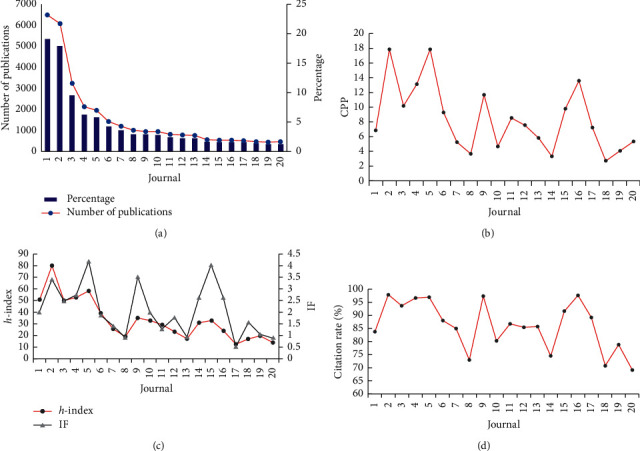
Top 20 journals according to numbers/percentage of publications (a), citations per paper (CPP) (b), *h*-index/IF (c), and citation rate (d) during 2009–2018. (1) Evidence-Based Complementary and Alternative Medicine. (2) Journal of Ethnopharmacology. (3) BMC Complementary and Alternative Medicine. (4) Planta Medica. (5) Phytomedicine. (6) The Journal of Alternative and Complementary Medicine. (7) Chinese Journal of Integrative Medicine. (8) Journal of Traditional Chinese Medicine. (9) The American Journal of Chinese Medicine. (10) Complementary Therapies in Medicine. (11) Journal of Manipulative and Physiological Therapeutics. (12) Chinese Journal of Natural Medicines. (13) European Journal of Integrative Medicine. (14) Integrative Cancer Therapies. (15) Journal of Ginseng Research. (16) Acupuncture in Medicine. (17) Boletin Latinoamericano y del Caribe de Plantas Medicinales y Aromaticas. (18) Complementary Therapies in Clinical Practice. (19) Explore: The Journal of Science and Healing. (20) Holistic Nursing Practice. England: 1, 3, 10, and 16. Ireland: 2. Germany: 4, 5, and 13. USA: 6, 9, 11, 14, 19, and 20. Mainland China: 7, 8, and 12. South Korea: 15. Chile: 17. Netherlands: 18.

Figure 5(a) shows the top 20 journals with the highest number of publications. The top 20 journals published 31667 papers in 2009–2018, which represents 92.9% of total publications in the field of ICM. The top journals were based in England, Ireland, Germany, the USA, China, South Korea, Chile, and the Netherlands. *Phytomedicine* had the highest CPP (Figure 5(b)).

Table 1 shows the impact factors of the top 20 journals. Comparing these impact factors over the past five years with current impact factors, we found that, as the volume of journal publications increased significantly, journal impact factors decreased. Only *Phytomedicine* (5), *The American Journal of Chinese Medicine* (9), and *Acupuncture in Medicine* (16) have improved their impact factors significantly.

Half of the top 20 journals only belong to the ICM category, and half belong to multiple categories. Most of the multicategory journals belong to the categories of Pharmacology & Pharmacy and Plant Sciences. The journals' names listed in Table 1 also show that the ICM field is closely related to the plants and ethnopharmacology.

*Evidence-Based Complementary and Alternative Medicine* was an open-access (OA) journal, ranking first in number of publications. In general, the IF values of journals in the ICM field are not high, relative to other medical fields. In the field of ICM, *Phytomedicine* had the highest IF, at only 4.18, while the IF for most journals in the ICM field was between 1 and 2. Although mainland China contributed the greatest number of publications, only three SCI-indexed journals are issued from mainland China: *Chinese Journal of Natural Medicines*, *Phytomedicine*, and *The Journal of Alternative and Complementary Medicine*.

The *h-*index (Hirsch index) [19] is an established indicator of the impact of an object (journal, author, topic, and institute) and is a parameter that synthesizes a researcher's output. It is defined as the maximum number, *h*, such that the researcher has *h* papers with at least *h* citations each [20]. It combines the effect of “quantity” (number of publications) and “quality” (citation rate) in a rather specific balanced way [21]. Analysis of the correlation between journal IF and *h*-index (*r* = 0.697) indicated that highly cited papers may contribute substantially to increases in journal IF (Figure 5(c)). Similarly, there was a positive correlation between journal IF and citation rate (*r* = 0.913), indicating that top journals are less likely to publish papers with no citations (Figure 5(d)).

### 3.4. Comparison of Keywords between the Top 500 Highly Cited and Noncited Papers

Keywords are the most condensed scientific components of a paper and can thus be used to trace hot topics and frontiers in specific research fields. As papers contributing to hot topic research and cutting-edge research are more likely to be highly cited,it is a reasonable hypothesis that keywords could differ markedly between highly cited and noncited papers. Keywords were retrieved from the top 500 highly cited and noncited papers in the field of ICM. Subsequent analysis showed that, from the top 500 highly cited papers, “in vitro,” “oxidative stress,” “mice,” and“antibacterial activity” were the most frequently used keywords plus, and “medicinal plants,” “antioxidant,” “anti-inflammatory,” “apoptosis,” and “ethnobotany” were the most frequently used author keywords. There were some differences between the two groups, but they were not substantial. Because in noncited papers, “expression,” “care,” “disease,” “prevalence,” “apoptosis,” “health,” “cancer,” “treatment,” “oxidative stress,” and “acupuncture” were the most frequently used keywords. From a scientific perspective, many of the keywords used in noncited papers pointed towards broad meaning that poorly reflect the exact research content. At the same time, comparing the keywords of highly cited and noncited papers, it can be seen that the keywords of highly cited papers directed to the pharmacological effects of medicinal plants.

### 3.5. Analysis of ESI Papers

Highly cited papers: selected from the most recent 10 years of data, highly cited papers reflect the top 1% of papers by field and publication year. Hot papers: hot papers are selected by virtue of being cited among the top one-tenth of one percent (0.1%) in a current bimonthly period. ESI categorizes papers into 22 broad fields of research. Papers are selected in each of 22 fields of science and must be published within the last two years. Highly cited papers help identify breakthrough research within a research field and are used within Web of Science to identify and refine the most influential research papers. ESI highly cited/hot papers are indicators of cutting-edge research. Notably, ICM is not an independent field in ESI, but rather is included into other ESI fields, according to the scientific characteristics of papers. Among the total of 34102 publications in ICM between 2009 and 2018, there were only 48 ESI highly cited/hot papers: 37 in Pharmacology and Pharmacy, 39 in Plant Sciences, 39 in Chemistry Medicinal, and 1 in Oncology (these papers were interdisciplinary). The 48 papers included 37 reviews and 11 research articles, with 15 OA and 33 non-OA papers. 15 of them belonged to international cooperation publications. 47 were highly cited papers, and 1 was hot paper (search in November 2019). The proportion of international cooperation papers in the top 500 papers was 21.6% and was 18.68% in all papers. The proportion of international cooperation papers in ESI highly cited/hot papers was 31.25%. It is not difficult to infer that international cooperation will help publications get more citations.

The *African Journal of Traditional Complementary and Alternative Medicines* and *Alternative Medicine Review* each had a highly cited article but were eliminated from SCI in 2018.

Table 2 lists the top 20 highly cited publications. Analysis of these highly cited publications demonstrated that, except for one industry standard, all others were related to the antioxidant and anti-inflammatory effects of plant extracts. Most reviews were related to the effects of natural plant extracts, while several were about ethnopharmacology and meta-analysis of integrative medicine data. Notably, four of the 37 reviews were related to ginsenosides. Given the important role of ginseng in Chinese medicine, scientists in the field of Chinese medicine are exploring its potential from a modern perspective.

The countries have the ESI papers were mainland China (15), South Korea (8), Italy (5), Switzerland (4), and the United Kingdom (4).

### 3.6. Analysis of OA Publications

**Figure 6:**
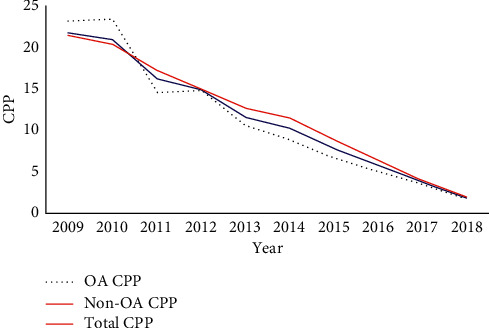
Comparison of the impact open access (OA), non-OA, and total ICM publications.

OA refers to online publications that are free of restrictions on access; hence, OA is expected to maximize the circulation and impact of research publications [22, 23]. Therefore, an increasing number of journals persuade, or even demand, that authors select OA for their papers. Accordingly, the number of ICM-related OA publications increased from 344 in 2009 to 1594 in 2018, representing a five-fold increase. Average citations of OA papers did not differ significantly from those of non-OA papers. Unexpectedly, the average citations of OA papers were lower than those for total papers and non-OA papers from 2009 to 2018 (Figure 6). This result indicates that OA may not be an effective approach to increase the citation of papers in the field of ICM.

## 4. Discussion

### 4.1. China Is Active in ICM Research and Remains with the Tremendous Potential

Our results demonstrate that Asia, and particularly mainland China, leads the world in ICM research. As China has over 5000 years of experience in the use of TCM, this is not surprising. Despite the rapidly development of western medicine in China, ICM remains an indispensable therapeutic approach, particularly for patients with chronic and complex diseases, such as cardiovascular and cerebrovascular diseases, metabolic syndromes, and cancer, among others. Scientific research into ICM in China can be traced back to the beginning of the last century. The achievements of ICM research in the past century include the successful identification of artemisia and its development to an effective antimalaria drug, and because of this great contribution, Professor Tu was awarded a Nobel Prize in 2015. Additionally, the identification of arsenic trioxide as an effective drug therapy for leukemia represents another great achievement of ICM research in China. In the 1980s, the Chinese government launched a long-term and extensive project, “modernization of TCM.” Since that time, China has made substantial financial investment in this area. In addition to the successful development of artemisia and arsenic trioxide as internationally recognized drugs, the successful entrance of Danshen dripping pills and other herbal preparations, into USA Food and Drug Administration, approved clinical trials represent another landmark in the achievements of “Modernization of TCM.”

The present bibliometric analysis clearly demonstrates that China is the leader in publication of ICM research, indicating the leading role of China in this field; however, it is also important to note that, based on CPP data, China is not preeminent, suggesting that the ICM publications from China have not received enough attention. WoS has an English-language bias. The problem cannot be ignored because myriad research findings of Chinese medicine, Islamic medicine, and Kampo are written in their native languages, not in English. As ICM emphasizes non-Western conventional medicines, this English-language bias is an obstacle to the ability to reflect any developing trends in this field accurately [24]. At the same time, our research shows that the level of international cooperation of highly cited papers was relatively high. Improving the level of international cooperation in ICM may be an effective way to attract more attention.

### 4.2. Plant Medicine in the ICM Field Gets More Attention

Our research shows that most of highly cited papers were related to the effects of natural plant extracts. As Tu said, artemisinin, with its unique sesquiterpene lactone created by phytochemical evolution, is a true gift from old Chinese medicine. The route to the discovery of artemisinin was short compared with those of many other phytochemical discoveries in drug development. But this is not the only instance in which the wisdom of Chinese medicine has borne fruit [25].

The development of omics technologies and network pharmacology in recent years has offered an opportunity to bridge the gap between western medicine and TCM [26]. Intensive and comprehensive research and validation of the pharmacological mechanisms, targets, and signaling pathways involved in ICM remain very scarce and may be an important component of future ICM research.

Moreover, alongside increasing recognition of the limitations of the “one gene, one drug, one disease” philosophy for therapy of chronic and complex diseases, western scientists have called for a renaissance of TCM research. Thus, it is reasonable to expect that research into ICM from western countries will expand. Worldwide attention will doubtless facilitate the development of ICM. In the spirit of the recent recognition of Professor Tu's outstanding contributions and the tremendous potential of natural product-based medicines, the scientific community should strengthen collaborative research in various areas of natural medicine, to enhance scientific research quality.

### 4.3. ICM Journals Should Play an Active Role in Promoting the Development of ICM

In our research, we retrieved journals classified in the ICM category by WoS. Importantly, a large number of papers in this field, particularly high-impact papers, are not published in “ICM” journals, but rather in mainstream medical journals; however, as a unique part of medicine, relevant scientists have always emphasized that we must have reliable scientific evidence and respect the characteristics of traditional medicine. ICM needs its own professional journal to improve the rate of publication of quality articles. Improving the influence of ICM professional journals is very important to promote the development of ICM.

In bibliometric analysis, the capture of keywords is a useful approach to identify the cutting edge in a specific field. A scan of the recent ICM articles showed that most were limited to analysis of compounds, identification, and assessment of active components or simple analyses of the pharmacological, pharmacokinetic, or toxicological performance of herbal extracts, while assessment of drug efficacy was primarily limited to antioxidant, anti-inflammatory, and cytotoxic properties, which lack specificity, resulting in a low likelihood of discovery of novel drugs from natural and traditional Chinese medicines. The methods used in integrative medicine encompass a wide range of therapeutic modalities, among which acupuncture and Chinese herbal medicine are commonly used. Owing to great interest among the general public, integrative medicine has frequently featured in the media recently, and the phrase “the medicine of the future” has been coined [27]. Adjuvant therapy using ICM is developing rapidly; however, from a bibliometrics perspective, the related literature has yet to attract sufficient attention. Tam et al. analyzed the top 50 publications in the ICM field and found that no articles used an observational study design [28]. Physicians who practice complementary and alternative medicine must recognize the need to support their methods with scientific evidence relating to their effectiveness [29]. The number of ICM publications has experienced a period of rapid growth. With the development of ICM, we are glad to see that a panel of important clinical trials of ICM had been published in high-impact journals. Our research selected the journals of ICM category, and the relevant literatures have not been discussed but cannot be ignored. Thomas Kuhn, a highly significant philosopher of science of the 20th century, argued that science does not progress via a linear accumulation of new knowledge but rather undergoes periodic revolutions, also referred to as “paradigm shifts” [30]. Appropriate changes are key to the further development of ICM.

Few ESI highly cited/hot papers were captured, further demonstrating the lack of innovative studies in ICM. Nevertheless, the keywords captured from previous publications may not represent the “real” research highlights, considering keywords such as “rats” and “in vitro” are too general to indicate specific scientific aspects of the published studies. Most high-impact journals clearly demand that such void keywords should not be used in their guidance to authors. For journals in the ICM field, it is important to guide authors use specific keywords to highlight their investigations.

The overall low impact of research in the field of ICM is also evidenced by the general low impact factor of journals in this field. The *Journal of Ginseng Research* has the highest impact factor, at only 3.898, among journals in the field of ICM. In contrast, the field of western medicine includes many high-impact journals; for example, *The New England Journal of Medicine*, *JAMA*, and *The Lancet* and its sister journals. Although it is well-acknowledged that journal impact factors exhibit biases across different scientific fields, because total publications vary dramatically, generally low impact factors of journals in a certain scientific field indicates that the research in this field is less active than those with higher impact factors. It is understandable that the research scale, intensity, and comprehensibility of ICM, as alternative medicine, are much lower than those of the western medicine. Ling et al. believed that TCM possesses advantages over Western medicine in specific aspects at a certain stage of cancer treatment [31]; however, to expand the influence of ICM research, it is important to gather sufficient scientific evidence to support the advantage of ICM over the typical western medicine for the therapy of chronic diseases.

We expected the further reform and development of professional ICM journals to better lead the field.

### 4.4. OA Publication Is Not a “Passkey” to Improve the Impact of Papers Reporting Poor Science

OA, which is a product of the belief that free and wide access to publications may help to expand the scientific impact of publications, has greatly promoted journal publication. The emergence and rapid growth of mega journals are part of the result of this development; however, the impact of OA papers, as evidenced by the results of CPP analysis in our study, was lower than that of both total papers and non-OA papers, indicating that OA did not improve the impact of publications in the field ICM. For OA, the authors are required to pay article processing charges and have promised rapid peer review and immediate online publication after acceptance, which leads to the quality of peer review becoming an essential concern [32, 33]. After OA becomes a common publication type, manuscript quality remains the key to competition among scientific and technical journals; therefore, improvement of academic quality and ensuring publications presents a rational perspective deserve top priority. OA is not an appropriate approach for journals to improve business by sacrificing the scientific quality of publications.

## 5. Conclusion

Worldwide attention will doubtlessly facilitate the development of ICM, which could represent a challenge for ICM scientists. The continuous efforts of Chinese scientists in the field of ICM have made China the leader in ICM research publications; however, the quality of publications requires improvement. Keyword analysis of ICM publications reflects the main research content in this field, while highly cited papers reflect the hot topics. The plant pharmacology part of ICM gets more attention. China has a leading advantage in the field of natural medicine in ICM and should continue to tap the potential in this area. The number of ICM journals is not large, with a few journals accounting for more than half of the publications and citations in this field. Excellent professional ICM journals play an important role in the development of ICM. The development of the ICM field and improvement of the impact of ICM professional journals should be mutually reinforcing. The number of ICM publications has experienced a period of rapid growth; however, its impact remains relatively weak. Related research on ICM requires more valid evidence, and appropriate changes will be key to the further development of ICM. OA has been a hot topic in recent years; however, in our analysis of articles in ICM field journals from the WoS Core Collection, we found that the OA approach is not effective in promoting paper citations.

Overall, although publications in the ICM field have received widespread attention, their influence is relatively low. The field of ICM has great potential and we are motivated by Professor Yoyo Tu and looking forward with great anticipation for the future development of ICM.

## Figures and Tables

**Table 1:** Top 20 journals for ICM publications.

Rank	Journal	5-year impact factor	Journal impact factor	Journal normalized citation impact	Journal country/region
1	Evidence-Based Complementary and Alternative Medicine	2.328	1.984	1.00023	England
2	Journal of Ethnopharmacology	3.671	3.414	1.00001	Ireland
3	BMC Complementary and Alternative Medicine	2.82	2.479	1.00001	England
4	Planta Medica	2.74	2.746	1.00015	Germany
5	Phytomedicine	3.928	4.180	0.99988	Germany
6	Journal of Alternative and Complementary Medicine	1.814	1.868	1.00002	USA
7	Chinese Journal of Integrative Medicine	1.388	1.445	1.00027	Mainland China
8	Journal of Traditional Chinese Medicine	1.211	0.907	1.00031	Mainland China
9	American Journal of Chinese Medicine	2.952	3.51	1.00026	USA
10	Complementary Therapies in Medicine	2.452	1.979	1.00000	England
11	Journal of Manipulative and Physiological Therapeutics	1.758	1.274	1.00005	USA
12	Chinese Journal of Natural Medicines	1.995	1.773	0.99997	Mainland China
13	European Journal of Integrative Medicine	0.971	0.948	1.00005	Germany
14	Integrative Cancer Therapies	2.773	2.634	0.99989	USA
15	Journal of Ginseng Research	4.007	4.029	1.00010	South Korea
16	Acupuncture in Medicine	2.083	2.637	0.99966	England
17	Boletin Latinoamericano y del Caribe de Plantas Medicinales y Aromaticas	0.584	0.5	0.99768	Chile
18	Complementary Therapies in Clinical Practice	1.874	1.587	1.00024	Netherlands
19	Explore: The Journal of Science and Healing	1.331	1.037	0.99963	USA
20	Holistic Nursing Practice	1.005	0.888	0.99988	USA

**Table 2:** Journals publishing the top 20 highly cited papers.

Title	Citations	Publication type	Journal	Publication year
Synergy research: approaching a new generation of phytopharmaceuticals	510	Review	Phytomedicine	2009
Anti-inflammatory properties of curcumin, a major constituent of *Curcuma longa*: a review of preclinical and clinical research	476	Review	Alternative Medicine Review	2009
Mindfulness-based stress reduction for stress management in healthy people: a review and meta-analysis	476	Review	Journal of Alternative and Complementary Medicine	2009
Berberine and *Coptidis rhizoma* as novel antineoplastic agents: a review of traditional use and biomedical investigations	299	Review	Journal of Ethnopharmacology	2009
Propolis: is there a potential for the development of new drugs?	295	Review	Journal of Ethnopharmacology	2011
Pentacyclic triterpenes of the lupane, oleanane and ursane group as tools in cancer therapy	258	Review	Planta Medica	2009
How many cancer patients use complementary and alternative medicine: a systematic review and meta-analysis	255	Review	Integrative Cancer Therapies	2012
Goji (*Lycium barbarum* and *L*. *chinense*): phytochemistry, pharmacology and safety in the perspective of traditional uses and recent popularity	244	Review	Planta Medica	2010
Eugenol (an essential oil of clove) acts as an antibacterial agent against *Salmonella typhi* by disrupting the cellular membrane	240	Article	Journal of Ethnopharmacology	2010
Comparative antioxidant and anti-inflammatory effects of [6]-gingerol, [8]-gingerol, [10]-gingerol and [6]-shogaol	226	Article	Journal of Ethnopharmacology	2010
Rosenroot (*Rhodiola rosea*): traditional use, chemical composition, pharmacology and clinical efficacy	224	Review	Phytomedicine	2010
Aconitum in traditional Chinese medicine: a valuable drug or an unpredictable risk?	223	Review	Journal of Ethnopharmacology	2009
Extraction, isolation and characterization of bioactive compounds from plants' extracts	204	Review	African Journal of Traditional Complementary and Alternative Medicines	2011
Antioxidant activity, total phenolic and total flavonoid contents of whole plant extracts *Torilis leptophylla* L.	199	Article	BMC Complementary and Alternative Medicine	2012
Traditional Chinese medicine network pharmacology: theory methodology and application	191	Review	Chinese Journal of Natural Medicines	2013
*Lonicera japonica* Thunb.: ethnopharmacology, phytochemistry and pharmacology of an important traditional Chinese medicine	187	Review	Journal of Ethnopharmacology	2011
Anti-cancer natural products isolated from Chinese medicinal herbs	186	Review	Chinese Medicine	2011
Kudzu root: traditional uses and potential medicinal benefits in diabetes and cardiovascular diseases	176	Review	Journal of Ethnopharmacology	2011
Recent advances on *Ilex paraguariensis* research: minireview	163	Review	Journal of Ethnopharmacology	2011
Pomegranate peel and fruit extracts: a review of potential anti-inflammatory and anti-infective effects	163	Review	Journal of Ethnopharmacology	2012

## Data Availability

The data used to support the findings of this study are included within the supplementary information files.

## Supplementary Materials

Supplementary Materials Supplementary Table S1: the main data of the ESI papers assessed in the present study. Keywordtext (.txt) was downloaded from Web of Science.

## References

[1] Liem A., Rahmawati K. D. (2017). The meaning of complementary, alternative and traditional medicine among the Indonesian psychology community: a pilot study. *Journal of Integrative Medicine*.

[2] Hu X. Y., Lorenc A., Kemper K., Liu J. P., Adams J., Robinson N. (2015). Defining integrative medicine in narrative and systematic reviews: a suggested checklist for reporting. *European Journal of Integrative Medicine*.

[3] Huang Y. L., Zhou M. Q., Deng Q. Q., Zhang J., Zhou P. B., Shang X. G. (2015). Bibliometric analysis for the literature of traditional Chinese medicine in PubMed. *Scientometrics*.

[4] Gamus D. (2015). Advances in research of complementary and integrative medicine: a review of recent publications in some of the leading medical journals. *Harefuah*.

[5] Keshet Y., Attias S., Ben-Arye E., Shaham M., Grimberg O., Schiff E. (2013). Integrative complementary medicine for treatment of bariatric perioperative symptoms: patients’ experiences and staff evaluations. *Bariatric Surgical Practice and Patient Care*.

[6] Zyoud S. H., Al-Jabi S. W., Sweileh W. M. (2015). Scientific publications from Arab world in leading journals of integrative and complementary medicine: a bibliometric analysis. *BMC complementary and alternative medicine*.

[7] Moral-Munoz J. A., Carballo-Costa L., Herrera-Viedma E., Cobo M. J. (2019). Production trends, collaboration, and main topics of the integrative and complementary oncology research area: a bibliometric analysis. *Integrative Cancer Therapies*.

[8] Moral-Munoz J. A., Arroyo-Morales M., Piper B. (2018). Thematic trends in complementary and alternative medicine applied in cancer-related Symptoms. *Journal of Data & Information Science*.

[9] Zhu J., Zhai X., Chang Y. (2018). Bibliometric analysis of integrated complementary medicine research articles included in science citation index. *Bulgarian Chemical Communications*.

[10] Fu J. Y., Zhang X., Zhao Y. H., Huang M. H., Chen D. Z. (2011). Bibliometric analysis of complementary and alternative medicine research over three decades. *Scientometrics*.

[11] Moral-Munoz J. A., Cobo M. J., Peis E., Arroyo-Morales M., Herrera-Viedma E. (2014). Analyzing the research in integrative & complementary medicine by means of science mapping. *Complementary Therapies in Medicine*.

[12] Danell J. A. B. (2014). Reception of integrative and complementary medicine (ICM) in scientific journals: a citation and co-word analysis. *Scientometrics*.

[13] Ellegaard O., Wallin J. A. (2015). The bibliometric analysis of scholarly production: how great is the impact?. *Scientometrics*.

[14] O’Keeffe M. E., Hanna T. N., Holmes D. (2016). The 100 most-cited original articles in cardiac computed tomography: a bibliometric analysis. *Journal of Cardiovascular Computed Tomography*.

[15] Trapp J. (2016). Web of science, scopus, and google scholar citation rates: a case study of medical physics and biomedical engineering: what gets cited and what doesn’t?. *Australasian physical & engineering sciences in medicine*.

[16] Ding Z. Q., Ge J. P., Wu X. M., Zheng X. N. (2013). Bibliometrics evaluation of research performance in pharmacology/pharmacy: China relative to ten representative countries. *Scientometrics*.

[17] Ding Z.-Q., Zheng X.-N., Wu X.-M. (2012). Strategies for expanding the international influences of academic journals: an example from Chinese pharmaceutical journals. *Serials Review*.

[18] Waltman L., Schreiber M. (2012). On the calculation of percentile-based bibliometric indicators. *Journal Of the American Society for Information Science and Technology*.

[19] Hirsch J. E. (2005). An index to quantify an individual’s scientific research output. *Proceedings of the National Academy of Sciences of the United States of America*.

[20] Quesada A. (2011). Further characterizations of the Hirsch index. *Scientometrics*.

[21] Braun T., Glanzel W., Schubert A. (2006). A Hirsch-type index for journals. *Scientometrics*.

[22] Timsit J. F., Citerio G., Lavilloniere M. (2015). Determinants of downloads and citations for articles published in intensive care medicine. *Intensive Care Medicine Experimental*.

[23] Wang X. W., Liu C., Mao W. L., Fang Z. C. (2015). The open access advantage considering citation, article usage and social media attention. *Scientometrics*.

[24] Fan K. W. (2015). Bias and other limitations affect measures of journals in integrative and complementary medicine Ka-wai Fan, PhD. *Journal of the Medical Library Association JMLA*.

[25] Tu Y. Y. (2011). The discovery of artemisinin (qinghaosu) and gifts from Chinese medicine. *Nature Medicine*.

[26] He B., Zhang G., Lu A. P. (2015). Integrative network analysis: bridging the gap between western medicine and traditional Chinese medicine. *Journal of Integrative Medicine*.

[27] Brinkhaus B., Teut M., Girke M. (2009). Fall congress for integrative medicine—model for the future. *Deutsche medizinische Wochenschrift*.

[28] Tam W. W. S., Wong E. L. Y., Wong F. C. Y., Cheung A. W. L. (2012). Citation classics in the integrative and complementary medicine literature: 50 frequently cited articles. *European Journal of Integrative Medicine*.

[29] Dobos G. (2009). Integrative medicine–medicine of the future or “old wine in new skins?”. *European Journal of Integrative Medicine*.

[30] Kuhn T. S. (1962). *The Structure of Scientific Revolutions*.

[31] Ling C. Q., Yue X. Q., Ling C. (2014). Three advantages of using traditional Chinese medicine to prevent and treat tumor. *Journal of Integrative Medicine*.

[32] Xia J. (2014). An examination of two Indian megajournals. *Learned Publishing*.

[33] Nicholas D., Watkinson A., Jamali H. R. (2015). Peer review: still king in the digital age. *Learned Publishing*.

