# Supplementary material for: Publications in Integrative and Complementary Medicine: A Ten-Year Bibliometric Survey in the Field of ICM
Source: Evid Based Complement Alternat Med. 2020 Oct 6;2020:4821950. doi: 10.1155/2020/4821950 (PMC7559521; doi:10.1155/2020/4821950)
Supplement: Supplementary Materials — Supplementary Table S1: the main data of the ESI papers assessed in the present study. Keywordtext (.txt) was downloaded from Web of Science. [file 4821950.f1.docx]

|  | **Title** | **Journal** | **Publication type** | **Year** | **Times Cited** | **IF** |
| --- | --- | --- | --- | --- | --- | --- |
| 1 | Eugenol (an essential oil of clove) acts as an antibacterial agent against Salmonella typhi by disrupting the cellular membrane | Journal of Ethnopharmacology | Article | 2010 | 240 | 3.41 |
| 2 | Comparative antioxidant and anti-inflammatory effects of [6]-gingerol, [8]-gingerol, [10]-gingerol and [6]-shogaol | Journal of Ethnopharmacology | Article | 2010 | 226 | 3.41 |
| 3 | Antioxidant activity, total phenolic and total flavonoid contents of whole plant extracts Torilis leptophylla L | BMC Complementary And Alternative Medicine | Article | 2012 | 199 | 2.48 |
| 4 | Synergistic antibacterial effect of curcumin against methicillin-resistant Staphylococcus aureus | Phytomedicine | Article | 2013 | 127 | 4.18 |
| 5 | Antioxidant and intestinal anti-inflammatory effects of plant-derived coumarin derivatives | Phytomedicine | Article | 2014 | 100 | 4.18 |
| 6 | Salvia miltiorrhiza: Traditional medicinal uses, chemistry, and pharmacology | Chinese Journal of Natural Medicines | Article | 2015 | 87 | 1.77 |
| 7 | Antidiabetic effects of Morus alba fruit polysaccharides on high-fat diet- and streptozotocin-induced type 2 diabetes in rats | Journal of Ethnopharmacology | Article | 2017 | 42 | 3.41 |
| 8 | In vitro antioxidative and anti-inflammatory effects of the compound K-rich fraction BIOGF1K, prepared from Panax ginseng | Journal of Ginseng Research | Article | 2017 | 37 | 4.03 |
| 9 | Novel RAS inhibitor 25-O-methylalisol F attenuates epithelial-to-mesenchymal transition and tubulo-interstitial fibrosis by selectively inhibiting TGF-beta-mediated Smad3 phosphorylation | Phytomedicine | Article | 2018 | 27 | 4.18 |
| 10 | Recommended standards for conducting and reporting ethnopharmacological field studies | Journal of Ethnopharmacology | Article | 2018 | 21 | 3.41 |
| 11 | Curcumol induces cell cycle arrest in colon cancer cells via reactive oxygen species and Akt/ GSK3 beta/cyclin D1 pathway | Journal of Ethnopharmacology | Article | 2018 | 20 | 3.41 |
| 12 | Synergy research: Approaching a new generation of phytopharmaceuticals | Phytomedicine | Review | 2009 | 510 | 4.18 |
| 13 | Anti-inflammatory Properties of Curcumin, a Major Constituent of Curcuma longa: A Review of Preclinical and Clinical Research | Alternative Medicine Review | Review | 2009 | 476 | N/A |
| 14 | Mindfulness-Based Stress Reduction for Stress Management in Healthy People: A Review and Meta-Analysis | Journal of Alternative And Complementary Medicine | Review | 2009 | 476 | 1.87 |
| 15 | Berberine and Coptidis Rhizoma as novel antineoplastic agents: A review of traditional use and biomedical investigations | Journal of Ethnopharmacology | Review | 2009 | 299 | 3.41 |
| 16 | Propolis: Is there a potential for the development of new drugs? | Journal of Ethnopharmacology | Review | 2011 | 295 | 3.41 |
| 17 | Pentacyclic Triterpenes of the Lupane, Oleanane and Ursane Group as Tools in Cancer Therapy | Planta Medica | Review | 2009 | 258 | 2.75 |
| 18 | How Many Cancer Patients Use Complementary and Alternative Medicine: A Systematic Review and Metaanalysis | Integrative Cancer Therapies | Review | 2012 | 255 | 2.63 |
| 19 | Goji (Lycium barbarum and L-chinense): Phytochemistry, Pharmacology and Safety in the Perspective of Traditional Uses and Recent Popularity | Planta Medica | Review | 2010 | 244 | 2.75 |
| 20 | Rosenroot (Rhodiola rosea): Traditional use, chemical composition, pharmacology and clinical efficacy | Phytomedicine | Review | 2010 | 224 | 4.18 |
| 21 | Aconitum in Traditional Chinese Medicine-A valuable drug or an unpredictable risk? | Journal of Ethnopharmacology | Review | 2009 | 223 | 3.41 |
| 22 | EXTRACTION, ISOLATION AND CHARACTERIZATION OF BIOACTIVE COMPOUNDS FROM PLANTS' EXTRACTS | African Journal of Traditional Complementary And Alternative Medicines | Review | 2011 | 204 | N/A |
| 23 | Traditional Chinese medicine network pharmacology: theory methodology and application" | Chinese Journal of Natural Medicines | Review | 2013 | 191 | 1.77 |
| 24 | Lonicera japonica Thunb.: Ethnopharmacology, phytochemistry and pharmacology of an important traditional Chinese medicine | Journal of Ethnopharmacology | Review | 2011 | 187 | 3.41 |
| 25 | Anti-cancer natural products isolated from chinese medicinal herbs | Chinese Medicine | Review | 2011 | 186 | 2.27 |
| 26 | Kudzu root: Traditional uses and potential medicinal benefits in diabetes and cardiovascular diseases | Journal of Ethnopharmacology | Review | 2011 | 176 | 3.41 |
| 27 | Recent advances on Ilex paraguariensis research: Minireview | Journal of Ethnopharmacology | Review | 2011 | 163 | 3.41 |
| 28 | Pomegranate peel and fruit extracts: A review of potential anti-inflammatory and anti-infective effects | Journal of Ethnopharmacology | Review | 2012 | 163 | 3.41 |
| 29 | A review on the medicinal potentials of ginseng and ginsenosides on cardiovascular diseases | Journal of Ginseng Research | Review | 2014 | 152 | 4.03 |
| 30 | Life or death: Neuroprotective and anticancer effects of quercetin | Journal of Ethnopharmacology | Review | 2012 | 145 | 3.41 |
| 31 | Triptolide: Progress on research in pharmacodynamics and toxicology | Journal of Ethnopharmacology | Review | 2014 | 134 | 3.41 |
| 32 | A review on plant-based rutin extraction methods and its pharmacological activities | Journal of Ethnopharmacology | Review | 2013 | 126 | 3.41 |
| 33 | Network pharmacology-based prediction of the active ingredients and potential targets of Chinese herbal Radix Curcumae formula for application to cardiovascular disease | Journal of Ethnopharmacology | Review | 2013 | 124 | 3.41 |
| 34 | Alternative Antimicrobial Approach: Nano-Antimicrobial Materials | Evidence-Based Complementary And Alternative Medicine | Review | 2015 | 119 | 1.98 |
| 35 | A comprehensive review of the therapeutic and pharmacological effects of ginseng and ginsenosides in central nervous system | Journal of Ginseng Research | Review | 2013 | 119 | 4.03 |
| 36 | Natural Products for the Treatment of Type 2 Diabetes Mellitus | Planta Medica | Review | 2015 | 119 | 2.75 |
| 37 | Meta-analysis of the effect and safety of berberine in the treatment of type 2 diabetes mellitus, hyperlipemia and hypertension | Journal of Ethnopharmacology | Review | 2015 | 108 | 3.41 |
| 38 | The use of plants in the traditional management of diabetes in Nigeria: Pharmacological and toxicological considerations | Journal of Ethnopharmacology | Review | 2014 | 101 | 3.41 |
| 39 | Chemical diversity of ginseng saponins from Panax ginseng | Journal of Ginseng Research | Review | 2015 | 98 | 4.03 |
| 40 | Traditional usages, botany, phytochemistry, pharmacology and toxicology of Polygonum multiflorum Thunb.: A review | Journal of Ethnopharmacology | Review | 2015 | 94 | 3.41 |
| 41 | Traditional uses, botany, phytochemistry, pharmacology and toxicology of Panax notoginseng (Burk.) FH Chen: A review | Journal of Ethnopharmacology | Review | 2016 | 80 | 3.41 |
| 42 | Role of ginsenosides, the main active components of Panax ginseng, in inflammatory responses and diseases | Journal of Ginseng Research | Review | 2017 | 45 | 4.03 |
| 43 | Molecular understanding of Epigallocatechin gallate (EGCG) in cardiovascular and metabolic diseases | Journal of Ethnopharmacology | Review | 2018 | 26 | 3.41 |
| 44 | Phytochemistry, pharmacology, quality control and future research of Forsythia suspensa (Thunb.) Vahl: A review | Journal of Ethnopharmacology | Review | 2018 | 24 | 3.41 |
| 45 | Cannabis sativa: A comprehensive ethnopharmacological review of a medicinal plant with a long history | Journal of Ethnopharmacology | Review | 2018 | 21 | 3.41 |
| 46 | Anti-inflammatory effects of luteolin: A review of in vitro, in vivo, and in silico studies | Journal of Ethnopharmacology | Review | 2018 | 20 | 3.41 |
| 47 | Best practice in research: Consensus Statement on Ethnopharmacological Field Studies - ConSEFS | Journal of Ethnopharmacology | Review | 2018 | 16 | 3.41 |
| 48 | Pharmacological and medical applications of Panax ginseng and ginsenosides: a review for use in cardiovascular diseases | Journal of Ginseng Research | Review | 2018 | 16 | 4.03 |
